# RACK1 and IRE1 participate in the translational quality control of amyloid precursor protein in *Drosophila* models of Alzheimer’s disease

**DOI:** 10.1016/j.jbc.2024.105719

**Published:** 2024-02-02

**Authors:** Yu Li, Dongyue Liu, Xuejing Zhang, Suman Rimal, Bingwei Lu, Shuangxi Li

**Affiliations:** 1 Shandong Provincial Key Laboratory of Animal Cell and Developmental Biology, School of Life Sciences, Shandong University, Qingdao, China; 2 Department of Pathology, Stanford University School of Medicine, Stanford, California, USA

**Keywords:** proteostasis, amyloid precursor protein (APP), ribosome-associated quality control (RQC), RACK1, IRE1, ER, *Drosophila*

## Abstract

Alzheimer’s disease (AD) is a progressive neurodegenerative disorder characterized by dysregulation of the expression and processing of the amyloid precursor protein (APP). Protein quality control systems are dedicated to remove faulty and deleterious proteins to maintain cellular protein homeostasis (proteostasis). Identidying mechanisms underlying APP protein regulation is crucial for understanding AD pathogenesis. However, the factors and associated molecular mechanisms regulating APP protein quality control remain poorly defined. In this study, we show that mutant APP with its mitochondrial-targeting sequence ablated exhibited predominant endoplasmic reticulum (ER) distribution and led to aberrant ER morphology, deficits in locomotor activity, and shortened lifespan. We searched for regulators that could counteract the toxicity caused by the ectopic expression of this mutant APP. Genetic removal of the ribosome-associated quality control (RQC) factor RACK1 resulted in reduced levels of ectopically expressed mutant APP. By contrast, gain of RACK1 function increased mutant APP level. Additionally, overexpression of the ER stress regulator (IRE1) resulted in reduced levels of ectopically expressed mutant APP. Mechanistically, the RQC related ATPase VCP/p97 and the E3 ubiquitin ligase Hrd1 were required for the reduction of mutant APP level by IRE1. These factors also regulated the expression and toxicity of ectopically expressed wild type APP, supporting their relevance to APP biology. Our results reveal functions of RACK1 and IRE1 in regulating the quality control of APP homeostasis and mitigating its pathogenic effects, with implications for the understanding and treatment of AD.

Protein misfolding and aggregation are common pathogenic mechanisms in many neurodegenerative diseases. AD is a progressive neurodegenerative disorder that affects millions of people worldwide. Defects in the expression or processing of APP have been implicated as major causes of AD (1). APP is synthesized on ER membrane-bound ribosomes and later processed in the ER lumen where it undergoes post-translational modifications and folding (2). Normally, APP protein homeostasis is tightly modulated by posttranslational modifications and quality control mechanisms to ensure proper folding and trafficking. β-amyloid (Aβ) peptides are derived from APP through sequential proteolytic cleavage by β- and γ secretases. Aβ is prone to misfolding and aggregation that results in proteotoxicity. Extensive studies have focused on understanding the mechanisms of APP trafficking and degradation (3, 4), which are essential for preventing the aberrant accumulation or aggregation of Aβ peptides that are thought to contribute to the development of AD (5, 6, 7).

Cellular protein synthesis, folding, and degradation are kept at a homeostatic state to maintain proteostasis (8, 9). However, protein synthesis, folding, and maturation are intrinsically error-prone processes (10). Errors in these processes could compromise proteostasis and lead to aberrant protein aggregation that characterizes age-related neurodegenerative diseases (11, 12, 13). Cells employ an extensive signaling network to maintain proteome integrity. Translation control is a critical cellular mechanism that regulates the production of proteins in response to various cellular signals (14). Elicited by ribosome stalling, ribosome-associated quality control (RQC) has emerged as a key mechanism for maintaining the quality of protein synthesis and preventing the accumulation of proteotoxic proteins (15, 16, 17). ZNF598 is a major sensor for stalled and collided ribosomes (18, 19). It mediates ribosomal small subunit protein ubiquitination and then triggers the disassembly of 40S ribosomes. Nuclear export mediator factor NEMF, or Caliban (Clbn) in *Drosophila*, binds to the 60S subunit and catalyzes 60S-associated nascent polypeptide modification by adding C-terminal Ala and Thr residues (CAT-tails) in a non-templated manner. The E3 ubiquitin ligase LTN1 is recruited to ubiquitinate the aberrant nascent polypeptides and target them for proteasomal degradation (20, 21). RQC ensures the quality of protein synthesis by monitoring and eliminating defective ribosome-nascent chain complexes in stalled ribosomes. A failure of efficient RQC would elicit proteotoxic stress and cause neurological disorders (21, 22, 23, 24, 25, 26, 27, 28). We have previously demonstrated that ribosomes stall at the ER membrane during co-translational translocation of APP-C99. The RQC machinery is recruited to resolve stalled translation, preventing the accumulation of C99 with C-terminal extension (CAT-tail) and amyloid plaques formation (29). However, additional regulators required for preventing abnormal APP protein accumulation remain to be uncovered.

In addition to RQC, another cellular pathway regulating the co-translational quality control of nascent polypeptides associated with stalled ribosomes on the ER membrane is ER quality control. ER quality control systems, including the unfolded protein response (UPR), monitor newly synthesized proteins for proper folding, and distinguish between correct and incorrect protein conformations to ensure that aberrant proteins are not further processed along the secretory pathway (30, 31, 32). When misfolded proteins are detected, the ER quality control pathways will target them for clearance through the ubiquitin-proteasome system or other pathways (33, 34).

In this study, we generated two transgenic fly strains, expressing a mitochondrial-targeting deficient mutant APP and wild-type APP. The mitochondrial-targeting deficient mutant APP was intended to promote a more exclusive ER targeting of APP, as the predicted mitochondrial and ER targeting signals may compete during the intracellular sorting of APP. This mutant APP may add more burden to the co-translational quality control systems at the ER. Flies expressing mutant APP had locomotor activity deficits and shortened life spans. Strikingly, mutant APP distributed robustly within the ER and caused abnormal ER morphology. Genetic screening identified RACK1, and IRE1 as modulators of the proteotoxicity of both mutant APP and wild-type APP, suggesting that the quality control mechanisms uncovered with mutant APP are also applicable to wild-type APP. This is important as in most disease conditions APP is present with normal sequence. Further investigation demonstrated that the suppression of APP toxicity by the genetic manipulation of RACK1 and IRE1 was associated with reduced protein levels of ectopically expressed mutant or wild-type APP. Moreover, the effect of IRE1 on APP protein level was partially dependent on the RQC-related factor VCP/p97 and the E3 ubiquitin ligase Hrd1. Thus, our data demonstrate a crucial link between RQC pathway components and APP proteostasis, providing potential new therapeutic targets for ameliorating APP-associated neurodegeneration.

## Results

### A mitochondrial targeting deficient APP mutant localizes to ER and elicits aberrant ER architecture

**Figure 1:**
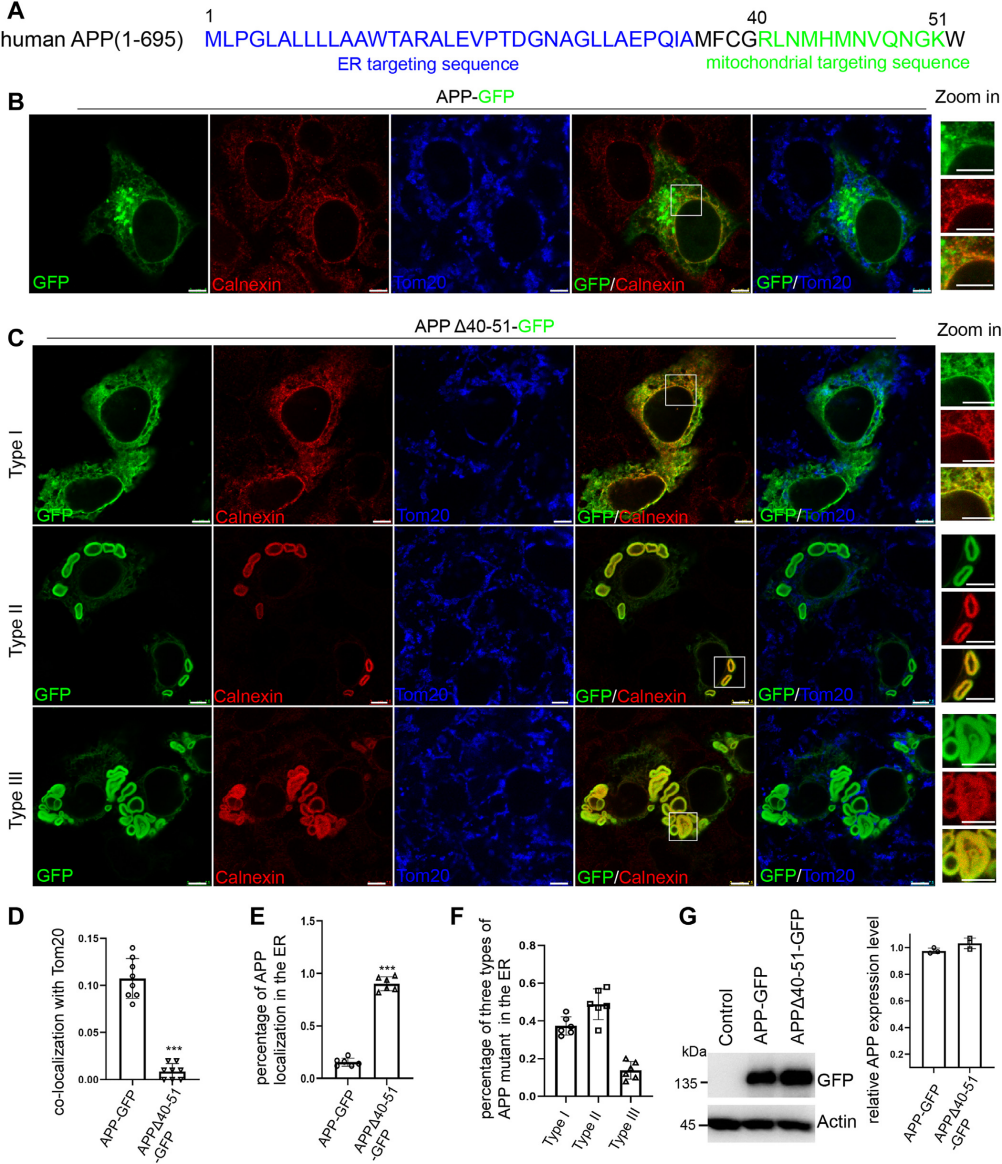
**Prominent ER localization of APP mutant in mammalian cells.***A*, the N-terminus of APP has putative dual ER and mitochondrial targeting sequences as shown in the schematic diagram. ER targeting sequences (1-36) are typed in *blue* and mitochondrial targeting sequences (40-51) in *green*. *B*, immunostaining of APP-GFP in HeLa cells. Calnexin in *red* is a marker for ER and Tom20 in *blue* a marker for mitochondria. APP was fused with GFP at the C terminus. Scale bars, 5 μm. *C*, representative confocal images of immunofluorescence of APPΔ(40--51)-GFP in HeLa cells exhibiting type I, type II and type III ER morphology. Scale bars, 5 μm. *D*, bar graph indicating the percentage of APP protein co-localizing with Tom20. ∗∗∗ *p* < 0.001 in Student’s *t* test. *E*, quantification of the proportion of normal and APP mutant distribution to the ER. *F*, quantification of the relative proportion of the three types of ER localization patterns induced by mutant APP protein. *G*, Western blot analysis showing APP-GFP and APP-Δ(40--51)-GFP expression level in HeLa cells. Bar graph shows the relative expression level of the GFP fusion proteins normalized to actin.

Mammalian APP is a type I transmembrane protein consisting of a large extracellular N-terminal domain, a transmembrane domain and a C-terminal cytoplasmic domain (35). There is a *Drosophila* APP homologue named APPL (36). Mammalian APP is synthesized and N-glycosylated in the ER and transported to the Golgi for maturation. Overexpression of APP was shown to induce defects in axonal transport (37, 38) and neurite outgrowth and arborization (39). Elucidating the molecular and cellular mechanisms of APP trafficking and metabolism within cells will contribute to our understanding of APP pathophysiology. To characterize specific regions important for its function, we first examined key amino acid sequences within human APP protein. We noticed that there are ER- and mitochondria-targeting sequences in the N-terminus of APP protein, suggesting APP protein can localize into both ER and mitochondria (Fig. 1*A*), consistent with previous observation of the dual-targeting of APP (40). We therefore made an APP deletion mutant construct APPΔ(40-51) by deleting the putative mitochondria-targeting sequence 40 to 51 (referred to as mutant APP here). To test whether mutant and normal APP behave differently in terms of localization, we performed immunostaining in HeLa cells by colocalization of GFP tagged mutant APP and wild type APP with the ER marker calnexin and mitochondrial marker Tom20. Wild type APP is known to have a variety of subcellular localizations, including the ER and endosomes (41), mitochondria (42) and Golgi apparatus (43). GFP-tagged wild type APP appeared to be associated with subcellular organelles (including the ER) in HeLa cells (Fig. 1*B*), whereas APPΔ(40-51) was more accumulated in the ER (Fig. 1*C*). Strikingly, we observed dramatic changes of ER morphology, including whorl-like ER structures, in mutant APP expressing cells, suggesting that mutant APP led to ER membrane remodeling. Correlating with the more attenuated mitochondrial localization of mutant APP compared to normal APP, as indicated by co-localization with the mitochondrial marker Tom20, increased distribution of mutant APP to the ER was observed (Fig. 1, *D* and *E*). These data suggested that mutant APP may fail to be exported out of the ER. Based on the changes of ER morphology, we classified them into type I, type II, and type III (Fig. 1*F*). Type I represented normal ER, type II contained concentric circular and enlarged ER, and type III had multilayered concentric ER whorls. Approximately 50% of mutant APP expressing cells displayed type II ER morphology and 15% with type III morphology. To exclude that the ER morphology alteration was dependent on APP protein level, Western blot analysis was carried out to confirm the comparable expression levels of mutant and normal APP in the cells analyzed (Fig. 1*G*). These results indicate that ectopic expression of mutant APP with the mitochondrial targeting sequence deleted profoundly altered ER morphology presumably due to its enriched accumulation in the ER.

### Expression of mutant APP causes abnormal ER morphology and behavioral deficits in flies

**Figure 2:**
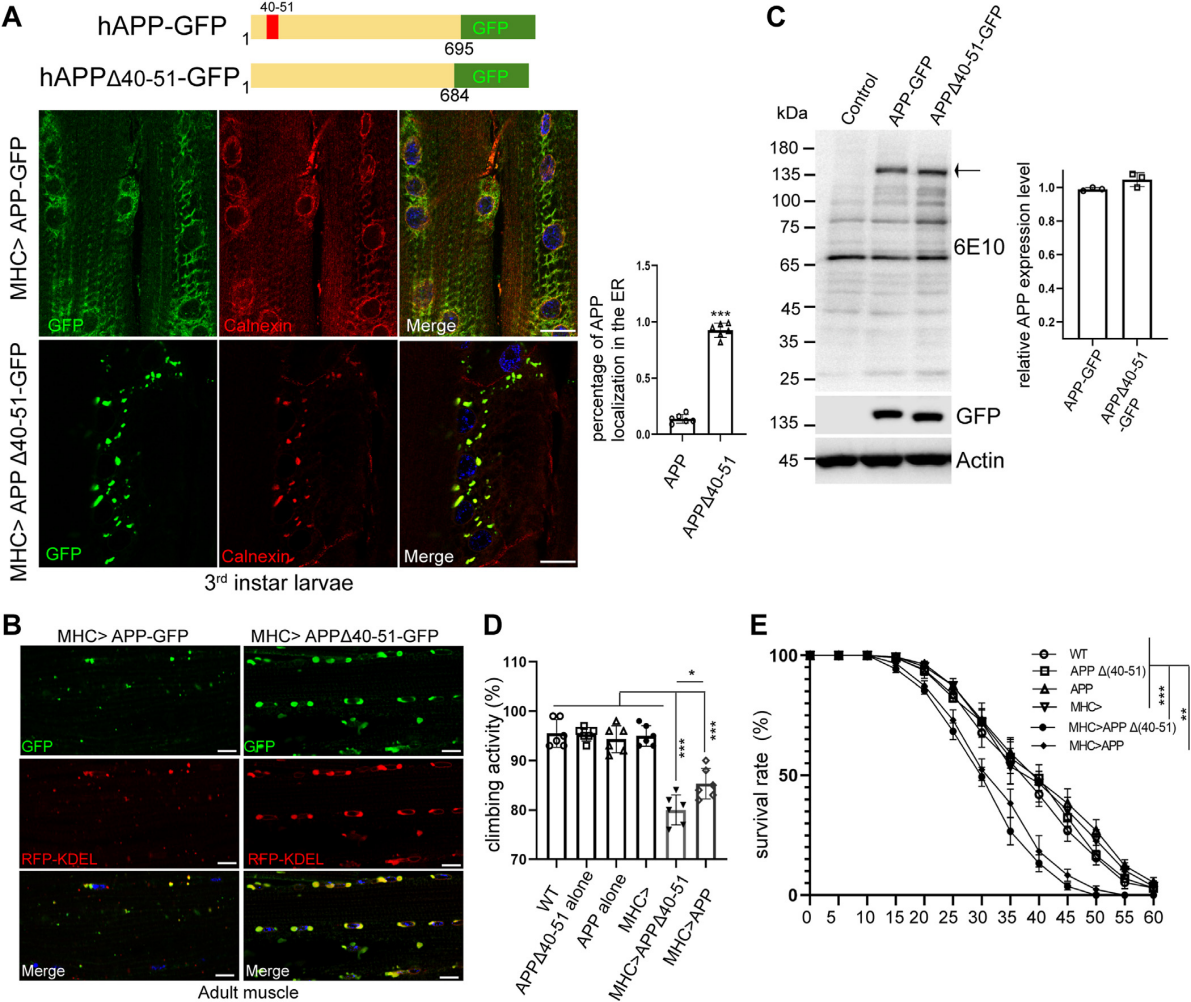
**The ER localization of APP in *Drosophila* results in defects in locomotor activity and lifespan.***A*, diagram showing wildtype and mutant APP constructs used for making transgenic flies. Images show immunostaining of *MHC>APP-GFP* and *MHC>APP-GFP Δ(40-51)* third instar larval muscle tissues. Calnexin is used to label ER. Regions of the sixth and seventh skeletal muscle fibers in segment four of third instar larval body wall muscle commonly used for neuromuscular junction (NMJ) studies were examined. Bar graph shows the percentage of APP distribution at the ER. Scale bars, 20 μm. Genotypes: *UAS-APP-GFP/+; MHC-GAL4/+* and *UAS-APPΔ(40-51)-GFP/+; MHC-GAL4/+*. *B*, validation of ER localization of APPΔ(40-51)-GFP in muscle tissues of 7-day-old adult flies. RFP-KDEL is used as marker for ER. Scale bars, 10 μm. Genotypes: *UAS-APP-GFP/+; MHC-GAL4/UAS-RFP-KDEL* and *UAS-APPΔ(40-51)-GFP/+; MHC-GAL4/UAS-RFP-KDEL*. *C*, Western blots of APP-GFP and APPΔ(40-51)-GFP expression level in 7-day-old adult fly muscle. The 6E10 antibody recognizes amino acid residue 1 to 16 of β-amyloid. Bar graph represents the relative level of APP expression. Genotypes: *MHC-GAL4/+* (control), *UAS-APP-GFP/+; MHC-GAL4/+* (APP-GFP), *UAS-APPΔ(40-51)-GFP/+; MHC-GAL4/+* (APPΔ(40-51)-GFP). *D*, effect of muscle expression of mutant and wild-type APP on locomotor activity of 25-day-old flies (n = 80). Animals eclosed within a 12 h window were aged for the study. Genotypes: *w*^*-*^ control (WT), *MHC-Gal4*/+ (MHC>), *UAS-APP-GFP* (APP alone), *UAS-APPΔ(40-51)-GFP* (APPΔ(40-51) alone), *MHC-GAL4>UAS-APP-GFP* (MHC > APP), *MHC-GAL4>UAS-APPΔ(40-51)-GFP* (MHC > APPΔ(40-51)). *E*, effect of muscle expression of mutant and wild type APP on life span (n = 90). Genotypes: *w*^*-*^ control (WT), *MHC-Gal4*/+ (MHC>), *UAS-APP-GFP* (APP alone), *UAS-APPΔ(40-51)-GFP* (APPΔ(40-51) alone), *MHC-GAL4>UAS-APP-GFP* (MHC > APP), *MHC-GAL4>UAS-APPΔ(40-51)-GFP* (MHC > APPΔ(40-51)).

The skeletal muscle is emerging as a primary tissue for studying disease pathogenesis that involves neuromuscular degeneration (44). It has been shown that the APP proteolytic product β-amyloid (Aβ) accumulates in skeletal muscle in Alzheimer's disease (45). To examine the *in vivo* effects of the mutant as well as normal APP, we cloned hAPPΔ(40-51)-GFP and hAPP-GFP into the *pUAST-attb* vector. Site specific attB/attP transgenes were generated by inserting the transgenes in the same chromosome site to minimize variability of expression levels due to positional effect of the transgenes. To test the assumption that wild type APP might also lead to ER morphology change *in vivo* when overexpressed, mutant and wild type APP *UAS* transgenes were expressed in muscle cells under the control of the muscle-specific *MHC>Gal4* driver. In third instar larval muscle cells, a small fraction of wild type APP protein was localized to the ER, as indicated by co-staining with the ER marker calnexin and data quantification. Consistent with the observation in cultured cells, mutant APP showed more prominent localization to the ER. Unlike in HeLa cells, mutant APP did not form whorl-like ER structure in fly muscle, with only enlarged ER puncta observed (Fig. 2*A*). We further validated that mutant APP exhibited abnormal ER accumulation by co-staining with the ER marker RFP-KDEL in adult muscle cells (Fig. 2*B*). Given that APP can be processed by proteolytic cleavages to generate Aβ-amyloid, we next asked whether wild type or mutant APP produced cleavage Aβ products. To address this, we performed Western blot analysis with the 6E10 antibody, which recognizes the C terminal sequence of APP. However, no significant amount of processed APP species was detected, suggesting that most of the wild type and mutant APP proteins were not subject to Aβ-amyloid like cleavage in fly muscle cells (Fig. 2*C*). While we cannot exclude the possibility that some small amount of processed APP species is causing toxicity, it is also possible that some aberrant FL-APP species stuck at the ER is causing toxicity by altering ER structure and function.

APP was previously shown to cause neuronal defects including reduced synapse number (46). To test the functional consequence of ectopic expression of mutant and wild-type APP, we evaluated locomotor activity and lifespan which were previously shown to correlate with muscle degeneration (29). Flies expressing mutant or wild-type APP in the muscle exhibited reduced climbing ability. Flies expressing mutant APP had more severe locomotor activity deficits, presumably because more mutant APP resided in the ER and led to disruption of ER protein homeostasis (Fig. 2*D*). Furthermore, while flies expressing wild type and mutant APP both exhibited shortened lifespan, mutant APP appeared to have stronger effect on lifespan (Fig. 2*E*). Thus, mutant APP exhibited ER distribution and proteotoxicity in *Drosophila* muscle cells, ultimately leading to abnormal ER morphology and impaired locomotor activity and lifespan.

### The RQC factor RACK1 is involved in the translational quality control of APP

**Figure 3:**
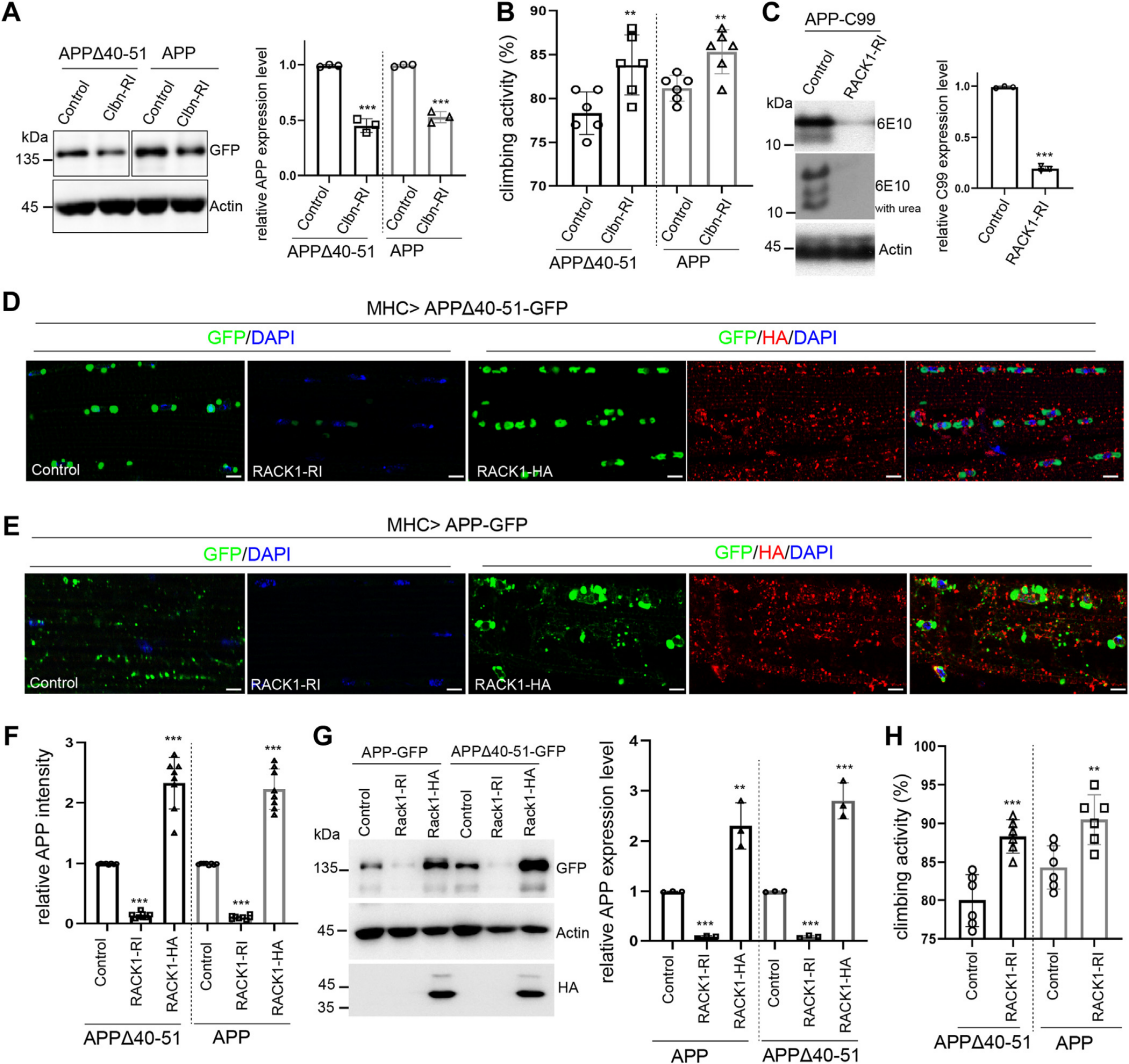
**The RQC factor RACK1 regulates mutant and wild type APP expression**. *A*, Western blot analysis and data quantification showing relative expression levels of mutant and wild-type APP upon knockdown of Clbn. *B*, effect of Clbn knockdown on locomotor activity of flies with muscle expression of mutant and wild type APP (n = 80). *C*, Western blot analysis and data quantification showing effects of RACK1 knockdown on APP-C99 protein level. *D*, immunostaining of APP in *Mhc>APP*Δ40 to 51 control flies or flies co-expressing RACK1-RI or RACK1-HA. Scale bars, 10 μm. Genotypes: *UAS-APPΔ40 to 51/+; MHC-GAL4/+* (control); *UAS-APPΔ40 to 51/+; MHC-GAL4/UAS-RACK1-RNAi* (RACK1-RI); and *UAS-APPΔ40 to 51/+; MHC-GAL4/UAS-RACK1-HA* (RACK1-HA). *E*, Immunostaining of APP in *Mhc>APP* control flies or flies co-expressing RACK1-RI or RACK1-HA. Scale bars, 10 μm. Genotypes: *UAS-APP/+; MHC-GAL4/+* (control), *UAS-APP/+; MHC-GAL4/UAS-RACK1-RNAi* (RACK1-RI), and *UAS-APP/+; MHC-GAL4/UAS-RACK1-HA* (RACK1-HA). *F*, bar graph showing quantification of APP intensity from adult fly muscle tissue. *G*, Western blot and data quantification showing relative expression level of mutant and wild type APP upon the knockdown or overexpression of RACK1. *H*, effect of RACK1-RI on climbing activity in flies with muscle expression of mutant or wild type APP (n = 80).

**Figure S2:**
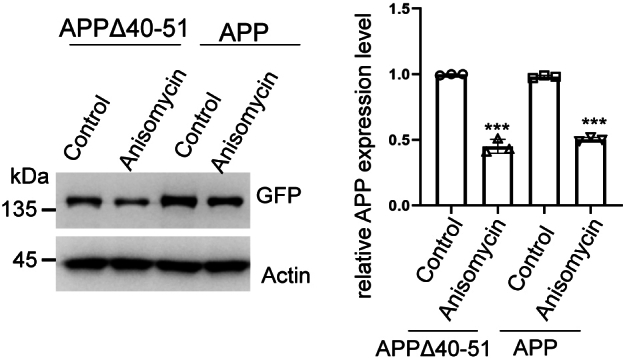
**Effects of ribosome stalling by anisomycin treatment on APP protein level.** Western blot indicates the relative expression level of mutant and wildtype APP upon anisomycin treatment. Bar graph shows the quantification of relative APP expression normalized to actin. ∗∗∗*p* < 0.001 in Student’s *t*-test.

Translation of mRNAs into proteins is a fundamental process requiring ribosome subunits and associated proteins. Receptor for activated C kinase 1 (RACK1) is a highly conserved scaffold protein that is involved in multiple signal transduction events associated with ribosomes (47). Besides a cytosolic free form, RACK1 is present as a 40S-associated protein involved in translational control (48). RACK1 is required for internal ribosome entry site (IRES)-dependent translation in *Drosophila* and in human hepatocytes (49). As a core RQC component, RACK1 stabilizes disomes, providing an interface for recognition by the E3 ubiquitin ligase ZNF598. It can resolve poly(A)-induced ribosome stalling by regulating RPS2, RPS3, and RPS20 ubiquitylation (19). We have previously shown that ribosome stalling and collision events happen during APP translation. Inefficient RQC during APP synthesis generates CAT-tailed species that precipitate hallmarks of Alzheimer’s disease (29). NEMF/Clbn is the only known enzyme involved in CAT-tailing (20). We found that the expression levels of mutant and normal APP were decreased when Clbn was knockdown (Fig. 3*A*). We were not able to detect additional CAT-tailed full-length APP (FL-APP) band at above 100kD molecular weight range, presumably because the CAT-tails are very short and their effect on the MW of FL-APP would be negligible. The band we detected at FL-APP position could be a mixture of CAT-tailed and unmodified APP. The reduction of WT and mutant FL-APP level by the knockdown of Clbn suggested that CAT-tailing might stabilize FL-APP, as previously observed for APP-C99 (29) and GR80 (20). Consistently, levels of mutant and normal FL-APP were reduced by the treatment with anisomycin (Fig. S2), a drug that inhibits CAT-tailing. We next examined locomotor activity upon loss of Clbn. Our results showed that knockdown of Clbn partially restored locomotor activity in transgenic flies expressing wild type or mutant APP (Fig. 3*B*).

Ribosome stalling and subsequent ribosome collisions during translation elongation would activate the RQC machinery, ultimately leading to the degradation of the problematic nascent polypeptides. Translationally stalled C-terminal fragment of APP (APP-C99) is regulated by the RQC pathway (29). We next sought out to evaluate the effect of RACK1 on ectopically expressed APP-C99, mutant APP, and wild-type APP. Western blot results showed that the knockdown of RACK1 dramatically diminished APP-C99 protein level, including the stalled and CAT-tailed APP-C99 species (Fig. 3*C*). We also performed immunostaining to show that the deficiency of RACK1 resulted in reduction of mutant and wild type APP fluorescence intensities. On the other hand, overexpression of RACK1 enhanced the fluorescence intensities of mutant and wild type APP (Fig. 3, *D*–*F*). We further carried out Western blot analysis to validate these observations. Results showed that mutant and wild type APP expression were abolished by the knockdown of RACK1. By contrast, mutant and wild type APP abundance were significantly increased by RACK1 overexpression (Fig. 3*G*). In addition, ablation of RACK1 ameliorated the locomotor deficits in flies expressing mutant and wild type APP (Fig. 3*H*). These data support that RACK1 is involved in the quality control of APP during translation.

### IRE1 regulates the abundance of mutant and wild-type APP proteins

When the accumulation of deleterious proteins exceeds the handling capacity of the ER, it will trigger the UPR, a signaling pathway aimed at restoring ER homeostasis (50). The UPR involves three major branches: IRE1/Xbp1, ATF6, and PERK/ATF4. Upon detection of misfolded substrates in the ER lumen, the phosphorylation and dimerization of the transmembrane sensor IRE1 induces the unconventional splicing of *Xbp1* mRNA, removing a 26-nucleotide intron (23 in flies) and producing Xbp1-s (spliced) (51, 52, 53).

**Figure 4:**
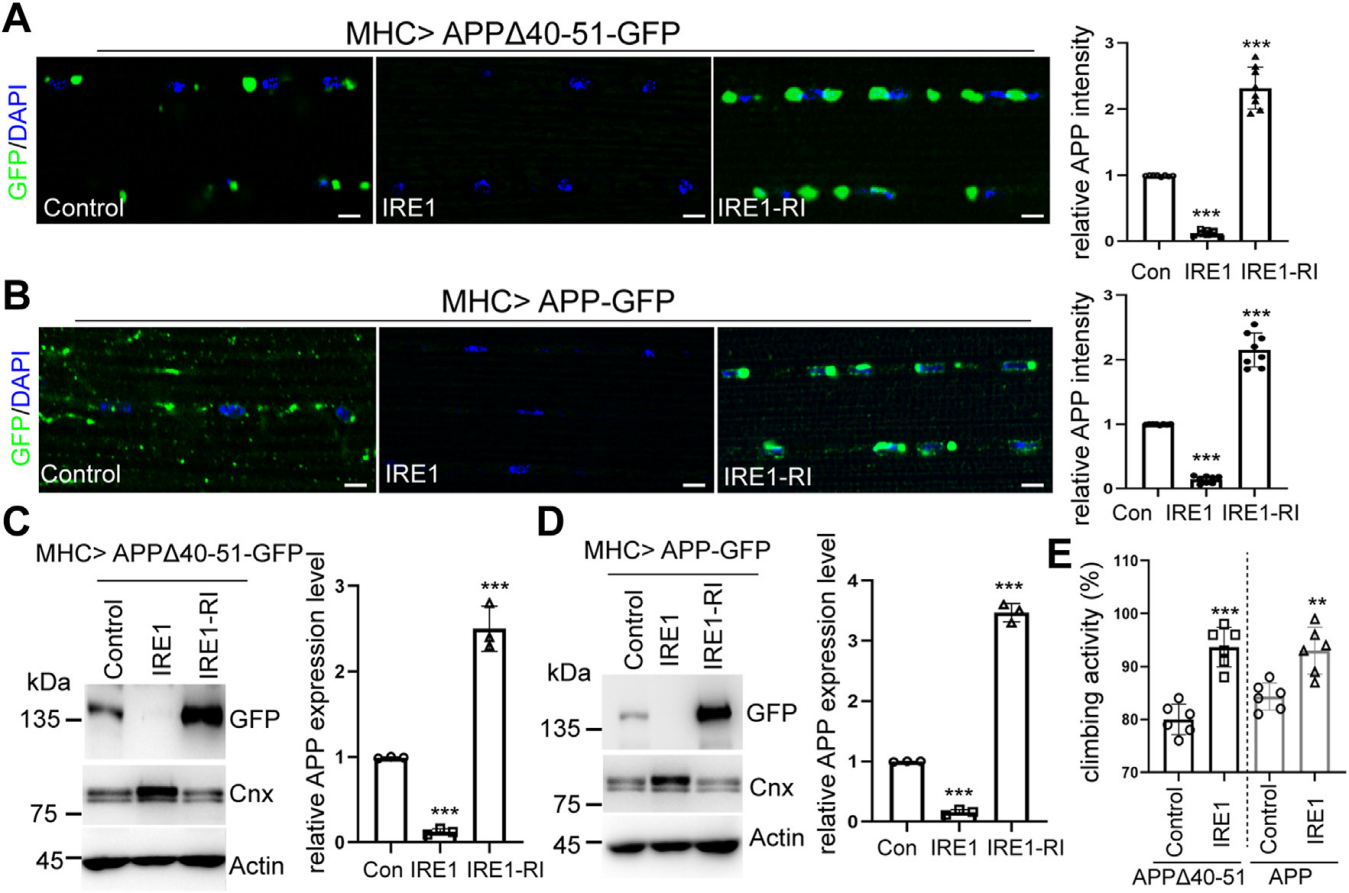
**IRE1 is a genetic modifier of APP-induced toxicity.***A*, representative immunostaining images and fluorescence intensity quantification showing the effect of IRE1 overexpression and IRE1-RI on APPΔ40 to 51 levels. Scale bars, 10 μm. *B*, representative immunostaining images and fluorescence intensity quantification showing effect of IRE1 overexpression and IRE1-RI on APP level. Scale bars, 10 μm. *C*, Western blot analysis showing effects of IRE1 overexpression or IRE1-RI on APPΔ40 to 51 and Calnexin expression levels. Bar graph shows quantification of relative APPΔ40 to 51 expression. *D*, Western blot analysis showing effects of IRE1 overexpression or IRE1-RI on APP and Calnexin expression level. Bar graph shows quantification of relative APP expression. *E*. climbing activity assay showing effects of IRE1 overexpression in mutant and wild type APP transgenic flies (n = 80).

**Figure S3:**
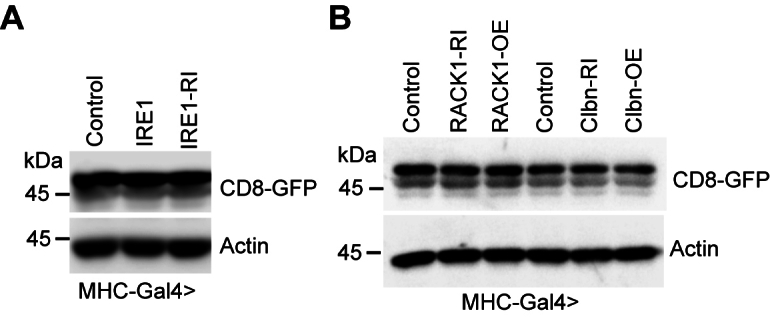
**Influences of quality control factors on ectopically expressed CD8 control type I transmembrane protein.***A*, Western blot data showing the effects of IRE1 overexpression and knockdown on type-I membrane protein CD8. *B*, Western blot data shows the expression level of CD8-GFP upon overexpression or knockdown of Clbn and RACK1.

Given that both the mutant and wild-type APP distribute to the ER and may result in ER stress, we tested if genetic gain or loss of IRE1 function would affect APP-induced toxicity. To address this, we co-expressed IRE1 overexpression or RNAi transgenes together with mutant or wild-type APP. Immunostaining results showed that ectopic expression of IRE1 significantly diminished mutant APP and wild-type APP fluorescence intensities, whereas the knockdown of IRE1 had opposite effects (Fig. 4, *A* and *B*). We further performed Western blot analysis to confirm the effect of IRE1 on APP protein level. Overexpression of IRE1 dramatically reduced APP and APPΔ(40-51) protein levels. By contrast, knockdown of IRE1 elevated APP and APP Δ(40-51) protein levels, suggesting that IRE1 plays critical roles in maintaining APP homeostasis (Fig. 4, *C* and *D*). To exclude the possibility that altered UPR component IRE1 might suppress global translational efficiencies, we examined the level of ER-specific type I transmembrane protein Calnexin (54). There was no change in Calnexin protein level upon IRE1 overexpression (Fig. 4, *C* and *D*). In addition, we validated that IRE1 had no effect on an ectopically expressed control type-I membrane protein CD8 (Fig. S3*A*). CD8-GFP expression level was also not affected by the genetic manipulations of Clbn and RACK1(Fig. S3*B*). At the functional level, gain of function of IRE1 mitigated locomotor deficits caused by mutant or wild-type APP overexpression (Fig. 4*E*). Together, these results suggest that IRE1 regulates the quality control of APP and modulates APP-induced toxicity at the whole animal level.

### IRE1 regulates the quality control of APP through the RQC pathway

**Figure 5:**
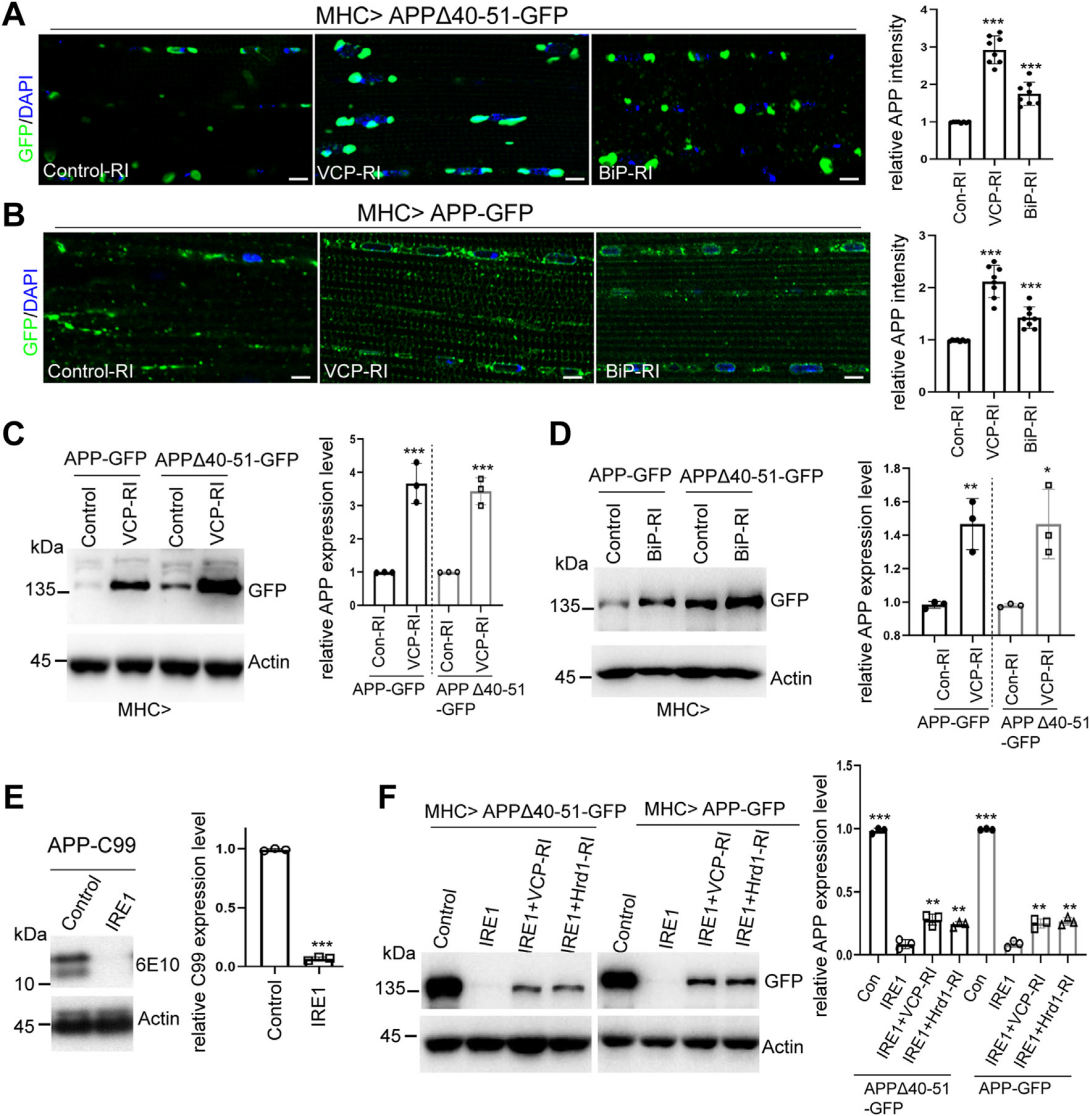
**The effect of IRE1 on APP requires the RQC component VCP and the E3 ubiquitin ligase Hrd1**. *A*, immunostaining images and quantification of APPΔ40 to 51 signal intensity in *Mhc-Gal4>APPΔ40 to 51-GFP* flies co-expressing *UAS-VCP-RI* and *UAS-BiP-RI* transgenes. Scale bars, 10 μm. *B*, immunostaining images and quantification of APP signal intensity in *Mhc-Gal4>APP-GFP* flies co-expressing *UAS-VCP-RI* and *UAS-BiP-RI* transgenes. Scale bars, 10 μm. *C*, Western blot results showing effects of VCP-RI on APP and APP Δ(40-51) levels. Bar graph shows quantification of relative protein level. *D*, Western blot results showing effects of BiP-RI on APP and APPΔ(40-51) levels. Bar graph shows quantification of relative protein level. *E*, Western blot results and data quantification showing effects of IRE1 on APP-C99 protein level. *F*, Western blot results and data quantification showing effect of knockdown of VCP or Hrd1 on APPΔ40 to 51 and APP protein level inhibited by IRE1.

RQC is an emerging important step in proteostasis. We have previously established its connection to the quality control of stalled translation of APP in AD (29) and poly(GR) in C9-ALS/FTD (20). In C9-ALS/FTD, a Notch-VCP axis regulates the stalled translation of poly(GR). VCP/p97, a key component of the RQC complex, is involved in the removal of nascent polypeptide chains from stalled ribosomes and targeting them for degradation (55, 56). VCP is required for mediating the effects of Notch on poly(GR) translation and toxicity (20). We tested whether the effect of IRE1 on APP protein homeostasis was also mediated by the VCP/RQC pathway. Immunostaining and Western blot results showed that loss of VCP resulted in increased levels of mutant and wild-type APP proteins (Fig. 5, *A*–*C*). The ER chaperon BiP/GRP78 has been documented to recognize ribosome stalling at the STOP-codon site, inducing degradation of stalled substrates (57). To test whether BiP is required for APP quality control, we examined wild-type APP and APPΔ(40-51) levels when BiP was knocked down in flies. Immunostaining and Western blot results showed that APP and APPΔ(40-51) protein levels were elevated when BiP was knocked down (Fig. 5, *A*, *B*, and *D*). These data suggested that VCP and BiP are involved in the quality control of APP.

We previously showed that inadequacy of RQC activities results in the accumulation of translationally stalled APP-C99 that would cause proteostasis failure and AD pathogenesis (29). The IRE1-XBP1 pathway has also been reported to protect against APP toxicity in *Drosophila* (58). To determine whether IRE1 is involved in the quality control of APP-C99, we performed Western blot assay. As shown in Figure 5*E*, APP-C99 protein level was dramatically diminished by the overexpression of IRE1. In the process of membrane protein biogenesis, after translocation into the ER, the ER quality control machinery assists in protein folding and facilitates the degradation of deleterious substrates. The ER resident E3 ligase Hrd1 was previously reported to be involved in the quality control of APP (59). We therefore tested whether VCP and Hrd1 may mediate the effects of IRE1 in APP regulation. We found that the knockdown of VCP and Hrd1 resulted in partial rescue of the attenuated APP protein level by IRE1 (Fig. 5*F*), supporting that VCP and Hrd1 mediate the effect of IRE1 in the quality control of APP translation and homeostasis.

**Figure 6:**
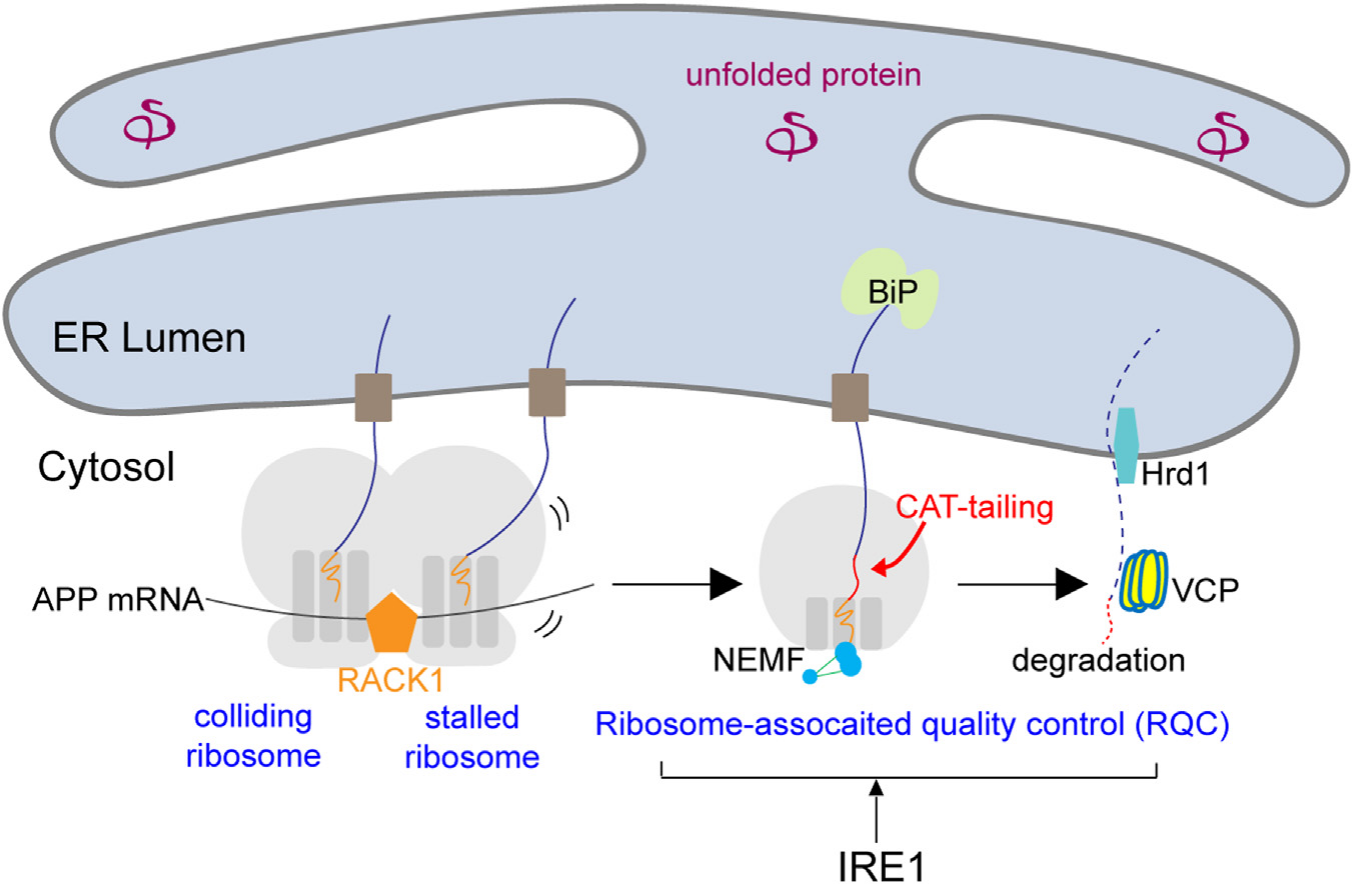
**A working model.** Schematic diagram illustrating the mechanisms by which RACK1 and IRE1 influence the quality control of APP during translation through RQC. RACK1 may directly act in the RQC process. IRE1 may act at upstream steps of the RQC pathways to regulate the turnover of mutant and normal APP proteins.

Our study identifies RACK1 and IRE1 are key modulators of the quality control of AD-associated APP. Two factors are closely linked to the RQC pathway for the removal of aberrant nascent polypeptides. RACK1 identifies ribosome collision events happening during *APP* translation and recruits RQC complexes to split the collided ribosomes. The ER chaperon BiP recognizes stalled APP nascent peptides from the ER lumen side and may facilitate the CAT-tailing of APP/APP-C99 by NEMF/Clbn. VCP may be involved in the extraction of stalled APP from the 60S ribosome. When APP or APP-C99 crosses the ER translocon and accumulates in the ER, VCP, and the E3 ubiquitin ligase Hrd1 may also cooperate to trigger APP removal through ERAD to maintain ER proteostasis. Therefore, our study shed light on a network of cellular mechanisms protecting against the deleterious accumulation of APP protein (Fig. 6).

## Discussion

APP is a transmembrane protein that undergoes extensive post-translational modifications and cleavage by several enzymes to generate amyloid-beta (Aβ) fragments, which can lead to the formation of amyloid aggregates, in turn causing proteostasis failure and cell death. Previous studies have largely focused on these post-translational events. Recent studies indicate that the co-translational RQC pathway is also critical in APP regulation (29). Initially found in yeast, RQC is emerging as a key quality control system for proteostasis. Mutations in RQC components Ltn1, HBS1, and NEMF are shown to cause neurodegenerative phenotypes in mouse models (60, 61, 62). Failure of proteostasis and cellular proteotoxic stress may cause profound ER membrane remodeling. Our study demonstrates that ER morphology becomes significantly altered when ER is overloaded with mutant APP protein. We uncover two important factors RACK1 and IRE1 that may induce the removal of APP resulting from ribosome stalling and collision during translation, prior to completion of the synthesis of FL-APP. RACK1 hyperactivation or deficit of IRE1 would impair cellular APP proteostasis, resulting in APP related proteotoxicity. This study highlights the importance of the RQC machinery in APP proteostasis. Clearance of toxic proteins during translation elongation therefore represents a feasible strategy for preventing aberrant protein accumulation in neurodegenerative diseases.

Recent evidence supported the relationship between the UPR and neurodegenerative diseases. As one major branch of the UPR, the IRE1/Xbp1 pathway has been extensively studied, but its role in disease remains uncertain. Enhancement of XBP1 was shown to protect against Parkinson’s disease (63). On the other hand, inhibition of IRE1 in cultured cells resulted in the degradation of APP by the proteasome and reduced AD-related pathology (64). In this study, we focused our analysis on muscle tissue since skeletal muscle has proven to be a suitable system for studying mechanisms of neuromuscular degeneration (44). In this system, activation of IRE1 dramatically reduced APP protein level, suggesting a protective role against APP by the IRE1 axis in fly muscle that differs from other models. Also in this fly muscle system, RACK1 and IRE1 participate in the regulation of APP protein homeostasis. Activation of IRE1 can significantly reduce APP abundance and alleviate APP-induced locomotor deficit. Loss of VCP/p97 or Hrd1 partially reversed the APP reduction conferred by IRE1, implicating VCP/p97 and Hrd1 in mediating the effect of IRE1 in the regulation of APP homeostasis. Future studies will investigate the molecular mechanisms linking IRE1 to the RQC pathway by VCP/p97 and Hrd1 and whether targeting these newly identified players may offer therapeutic benefit against AD in mammalian models.

## Experimental procedures

### Fly genetics

Fly stocks were purchased from the Bloomington, FlyORF, and VDRC *Drosophila* stock centers. The VCP-RI (V#24354; BL#32869), Clbn-RI (BL#62402), BiP-RNAi (V#14882), Hrd1-RI (BL#61344), RACK1-RI (BL#34694), RACK1-OE (FlyORF#F001043), IRE1-RI (BL#62156), IRE1-RI (V#39561), mCD8-GFP (BL#5137), and RFP-KDEL (BL#30909) were obtained. The *IRE1* (65) line was obtained from Dr Wei Song’s lab (Wuhan University). To generate *UAS-APPΔ(40-51)-GFP* and *UAS-APP-GFP* transgenic flies, the pUAST-attb-APPΔ(40-51)-GFP and pUAST-attb-APP-GFP constructs were used to generate transgenic flies by the Facility of *Drosophila* Resource and Technique (Institutes of Biochemistry and Cell Biology) using standard germline transformation procedures. Flies were maintained at 25 °C incubator on food containing Water, 17L; Agar, 93g; Cornmeal, 1716g; Brewer’s yeast extract, 310g; Sucrose, 517g; Dextrose, 1033g. *Drosophila* crosses were carried out with standard procedures under a 12 h light/dark cycle.

### Larvae muscle cell immunostaining

The standard neuromuscular junction (NMJ) analysis protocol was followed for the dissection of *Drosophila* larval muscle. Initially, third instar larvae were arranged in a Petri dish filled with PBS, positioned in an anterior to posterior orientation using two dissection pins. A midline incision was made in the larvae using dissection scissors, and the larval body wall was flattened and secured with four pins. The internal tissues were removed from the ventral side of the larvae using forceps, exposing the body wall muscles. The dissected larval muscle tissue was subjected to three washes with PBS before being fixed in Bouin’s solution for 5 min. Subsequently, the tissue was rinsed three times with PBST (PBS with 0.1% Triton X-100) for 15 min each and then incubated with a blocking solution at room temperature for 1 h. The muscle tissues were incubated with primary antibodies of interest at 4°C overnight with gentle shaking. After three washes, the muscle tissues were incubated with secondary antibodies for 2 h with shaking. Finally, the tissues were washed three times with PBST for 15 min each, mounted in Slowfade mounting medium, and covered with a coverslip. Visualization and imaging of the third instar muscle samples were performed using a ZEISS LSM900 confocal microscope.

### Western blot analyses

Adult muscle tissue or cultured cells were extracted using a lysis buffer consisting of 50 mM Tris-HCl (pH 7.4), 150 mM NaCl, 5 mM EDTA, 10% glycerol, 1% Triton X-100, and protease inhibitors. The lysate was then subjected to centrifugation at 10,000 g for 5 min, allowing the collection of the supernatant. This supernatant was further analyzed using SDS-PAGE and proteins transferred onto a PVDF membrane with a pore size of 0.45 μm (Millipore). The PVDF membrane was incubated with secondary antibodies Goat anti-Mouse IgG-HRP (Santa Cruz, sc-2005) or Goat anti-Rabbit IgG-HRP (Santa Cruz, sc-2004). Finally, the membrane was processed using a Konica Minolta SRX-101A medical film processor. Primary antibodies used for Western blot and their dilutions were: mouse anti-actin (1:5000, Sigma, A2228), mouse anti-GFP (1:3000, Proteintech, 66002-1-Ig), mouse anti-β-amyloid (clone 6E10) (1:500, Biolegend, SIG-39320), mouse anti-Cnx99A (1:400, DSHB), rabbit anti-HA (1:1000, Proteintech, 51064-2-AP). To better separate APP.C99 bands, fly thoracic muscle lysates were homogenized in lysis buffer containing 6M Urea. MES running buffer and 16% Tricine gel (Invitrogen #EC66955) were used for protein separation and western blotting.

### Fly muscle immunostaining

The muscle samples were fixed in a 4% formaldehyde solution at room temperature for 30 min. Subsequently, the samples were rinsed with PBST (PBS with 0.1% Triton X-100) for 4 times, 15 min each time. The samples were then treated with a blocking solution containing 5% BSA in PBS. Following this, the samples were incubated with primary antibodies overnight at 4 °C. Afterwards, the samples were washed again with PBST for 4 times, 15 min each time, and then incubated with secondary antibodies labeled with Alexa Fluor 568 or Alexa Fluor 488 (1:500 dilution, Invitrogen) for 2 h at room temperature. After washing with PBST for 4 times, 15 min each time, the samples were mounted using Slow-Fade mounting medium (Invitrogen) for imaging. The primary antibodies used in this study were chicken anti-GFP antibody (1:1000, Abcam, ab13970); mouse anti-HA antibody (1:1000, Santa Cruz, sc7392); mouse anti-Cnx99A antibody (1:100, DSHB). Representative images were shown for each genotype, with eight individual samples for each genotype analyzed. All tissue immunostaining images were obtained under identical gain settings as well as laser intensities.

### Mammalian cell culture and transfection

HeLa cells (ATCC) were maintained at 37 °C with 5% CO2 in Dulbecco’s modified Eagle’s medium–high glucose (DMEM, Sigma Aldrich) supplied with 10% fetal bovine serum (FBS). Plasmid transfection was carried out using Lipofectamine 3000 (cat#: L3000015, Invitrogen). After 72 h of transfection, cells were collected and subjected to immunostaining or Western blot analysis. 50 μM concentration of Anisomycin (A9789, Sigma) was used for treatment of cultured cells.

### DNA constructs

The APP-GFP plasmid (Addgene #69924) was purchased from Addgene. The APPΔ(40-51)-GFP deletion mutant was generated using the Q5 Site-Directed Mutagenesis kit (E0554, NEB) with the forward primer: TGGGATTCAGATCCATCAG and reverse primer: GCCACAGAACATGGCAAT. The *pUAST-attB-APPΔ(40-51)-GFP* and *pUAST-attB-APP-GFP* constructs were generated by inserting APPΔ(40-51)-GFP and APP-GFP into the NotI/XbaI restriction sites of pUAST-attB plasmid. All generated plasmids were verified by DNA sequencing.

### Climbing assay

After transferring 15 male flies to clean empty vial, the flies were given a period of 4 min to adapt to the new environment. After this adaptation period, the ability of the flies to climb vertically was evaluated in response to a bang stimulus in the negative geotaxis assay. The experiments were quantified by calculating the percentage of flies that managed to surpass a 4 cm height line within a 12s timeframe. Each experiment was conducted at least four times.

### Statistical analysis

Western blotting and immunostaining analysis were quantified by measuring relative intensities using the NIH Image J and GraphPad Prism eight software. Statistical evaluation was performed using Student’s *t* test and one-way ANOVA test. The results are displayed as mean ± SD, where ∗ represents *p* < 0.05, ∗∗ represents *p* < 0.01, and ∗∗∗ represents *p* < 0.001.

## Data availability

The authors confirm that all the data described in this study are contained within the article and its supplementary materials.

## Supporting information

This article contains supporting information.

## Conflict of interest

The authors declare that they have no conflict of interest.

## Figures and Tables

**Figure S1:**
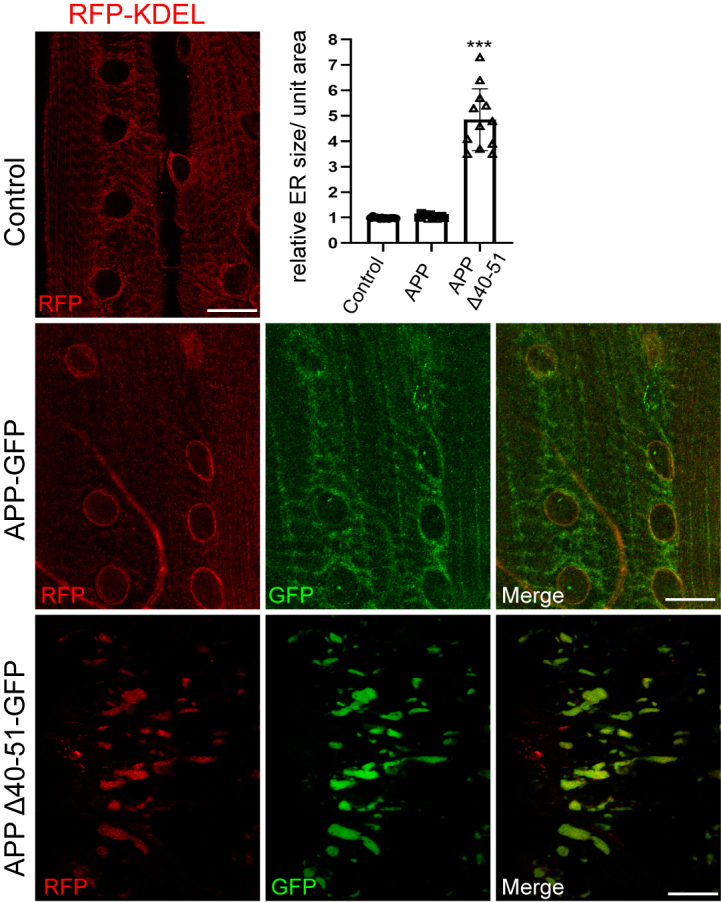
**Effect of ectopic expression of APP on ER morphology.** Images show RFP and GFP immunostainings and merged images of 3^rd^ instar level muscle tissues of *MHC-Gal4>RFP-KDEL*, *MHC-Gal4>RFP-KDEL, APP-GFP/+*, and *MHC-Gal4>RFP-KDEL, APPΔ(40-51)-GFP/+* animals. Scale bars, 20 μm. Bar graph shows quantification of the relative ER size per unit area. ∗∗∗*p* < 0.001 in Student’s *t*-test.
